# The effect of comprehensive rehabilitation program plus chemotherapy on quality of life in patients with postoperative non-small-cell lung cancer: study protocol of a multicenter randomized clinical trial

**DOI:** 10.1186/s13063-020-4162-1

**Published:** 2020-04-03

**Authors:** Jialin Yao, Lijing Jiao, Yiqing Yao, Yan Lu, Jun Shi, Jiaqi Li, Peiqi Chen, Ling Xu, Yabin Gong

**Affiliations:** 1 Department of Oncology, Yueyang Hospital of Integrated Traditional Chinese and Western Medicine, Shanghai University of Traditional Chinese Medicine, 110 Ganhe Rd, Shanghai, 200437 China; 2 Institute of Clinical Immunology, Yueyang Hospital of Integrated Traditional Chinese and Western Medicine, Shanghai University of Traditional Chinese Medicine, Shanghai, 200437 China; 3 Tumor Institute of Traditional Chinese Medicine, Shanghai Research Institute of Traditional Chinese Medicine, Shanghai, 200032 China

**Keywords:** Chinese herbal medicine, Liu Zi Jue exercises, Rehabilitation, Nonsmall-cell lung cancer, Quality of life

## Abstract

**Background:**

Comprehensive rehabilitation therapy based on traditional Chinese medicine (TCM) has been widely applied in various cancer treatments in China. Thus far, Chinese herbal medicine (CHM) has been shown effective in reducing the adverse effects of chemotherapy and improving the quality of life (QoL) during chemotherapy. The purpose of the present study is to compare the effects of CHM plus Liu Zi Jue (LZJ) exercises with CHM plus rehabilitation education and with placebo plus rehabilitation education in patients who have undergone complete resection for nonsmall-cell lung cancer (NSCLC) followed by postoperative adjuvant chemotherapy.

**Methods and design:**

A multicenter, randomized clinical trial will be performed with 354 stage Ib–IIIa NSCLC patients in five centers in China. Patients satisfying the inclusion criteria will be randomly divided into three groups according to a 1:1:1 ratio: intervention group A (IGA), intervention group B (IGB), and control group (CG). Each group will receive adjuvant platinum-based doublet chemotherapy for a total of four cycles. IGA participants will receive chemotherapy combined with CHM and LZJ exercises, IGB participants will receive chemotherapy combined with CHM and rehabilitation education, and CG participants will receive chemotherapy combined with placebo and rehabilitation education. The herbal treatment patients will be given granules daily and LZJ exercises will be performed four times per week during chemotherapy. The primary outcome is QoL, which will be assessed with the European Organization for Research and Treatment of Cancer (EORTC)-QLQ-C43 scale in each cycle. The secondary outcomes include the 2-year disease-free survival rate, disease-free survival, TCM symptoms, tumor markers, safety, and adverse events. After treatment, the patients will be followed up every 3 months within 2 years and every 6 months after 2 years until disease recurrence and/or metastasis.

**Discussion:**

Our previous study reported that CHM in combination with chemotherapy could lower the overall incidence of adverse events but increased digestive and gastrointestinal side effects compared with chemotherapy alone in postoperative NSCLC patients. This study will lay a foundation for the effectiveness of chemotherapy with or without a comprehensive rehabilitation program for QoL in patients with postoperative NSCLC.

**Trial registration:**

ClinicalTrials.gov, NCT03372694. Retrospectively registered on 17 December 2018.

## Background

Lung cancer is the leading cause of cancer death in China and worldwide [1, 2]. Nonsmall-cell lung cancer (NSCLC) accounts for nearly 92% of lung cancer, a significant increase over the past few years. Surgery is considered the best treatment for patients with early-stage lung cancer, yet a high risk of recurrence or metastasis remains. The 5-year survival rate for patients with stage IIIa NSCLC is less than 40% [3]. Cisplatin-based chemotherapy could reduce the risk of death by 13% and increase the 5-year survival rate by 5% in patients with completely resected NSCLC [4]. Accordingly, platinum-based adjuvant chemotherapy has been recognized as the standard treatment for patients with stage II–IIIa NSCLC [5, 6], and several studies have suggested that some stage Ib patients with a high-risk prognosis could consider chemotherapy [7].

However, the toxicity and side effects of chemotherapy can reduce the quality of life (QoL) of patients and even terminate chemotherapy. A total of 14% and 10% of the completely resected patients underwent only one and two cycles of cisplatin-based chemotherapy, respectively. Refusal, toxicity, and early death or progression were the major causes of termination. In total, 66% of NSCLC patients would endure grade 3–4 toxicity during adjuvant chemotherapy [8]. Although there were no differences in the QoL in lung cancer patients after lobectomy and sleeve resection, the JBR.10 study suggested that adjuvant chemotherapy had a direct negative effect on many aspects of QoL. For instance, the main symptoms are fatigue, loss of appetite, nausea, and vomiting. Due to the toxicity and side effects of platinum-based regimens, NSCLC patients after radical resections need a relatively slow recovery [9]. In the early postsurgery period (baseline to 3 months), more patients without chemotherapy had an improved QoL in global functioning [10]. However, receiving adjuvant chemotherapy can improve the long-term QoL of patients. The most vital goal for adjuvant chemotherapy is to extend the overall survival of early-stage cancers. Clinical oncologists and patients are beginning to accept the risks of some side effects for achieving survival benefits, whereas only 48–50% of patients could complete four cycles of chemotherapy [8]. Over 50% of NSCLCs are diagnosed in patients aged older than 65 years. Compared with young patients, fewer elderly patients completed treatment, and a great number refused treatment [11]. A considerable number of randomized controlled trials revealed that complementary therapy may solve numerous different problems in lung cancer patients (e.g., anxiety, pain, QoL, and treatment-related side effects). An evidenced-based approach to modern cancer care should combine complementary therapies with standard cancer therapies (e.g., surgery, radiation, and chemotherapy as well as the optimal supportive care measures) [12]. TCM, as one of the crucial components of complementary therapy for cancer, covers Chinese herbal medicine (CHM) and nondrug therapy (e.g., acupuncture, massage, and function rehabilitation.). Previous studies suggested that oral CHM can improve tolerance to chemotherapy, alleviate symptoms, and improve the QoL in lung cancer patients [13–17]. Liu Zi Jue (LZJ) exercises, a type of TCM health exercise, have been widely accepted in lung cancer patients. LZJ is employed to improve pulmonary function, regulate blood circulation, and improve physical fitness by six different pronunciations emitted during exhalation. Several clinical trials have verified that LZJ exercises are capable of significantly improving pulmonary function and QoL in patients carrying chronic obstructive pulmonary disease [18, 19]. It is expected that the combination of CHM and LZJ exercises will not only improve postoperative motor function in lung cancer patients but also reduce the toxicity resulting from postoperative adjuvant chemotherapy to successfully achieve four cycles of chemotherapy. Our previous study reported that the adverse events from CHM combined with chemotherapy had a lower overall incidence than those from chemotherapy alone, especially in the improvement of symptoms (e.g., pain, diarrhea, and hemoptysis). However, it was also observed that CHM could increase the incidence of nausea and vomiting in the combined chemotherapy [20].

Accordingly, it is recommended to suspend CHM on the day of chemotherapy and to take stomach-regulating medications within 1 week after chemotherapy. In addition, TCM syndrome differentiation medications should be given at 2–3 weeks. These medications help to improve the QoL and increase the passing rate of chemotherapy. From the first month to the second year after surgery, the QoL of NSCLC patients decreased significantly. The influencing factors included age, female sex, the increase in symptoms before and after surgery, the extent of surgical resection, and more postoperative complications [21–23]. Alleviating symptoms and improving the QoL are vital for the subsequent treatment of postoperative NSCLC patients.

Pulmonary rehabilitation is critical for the treatment of chronic lung diseases, which can improve exercise tolerance, relieve dyspnea, increase muscle strength, and improve health-related quality of life [24–28]. However, bicycle cycling with an increase in load, a frequently used method of pulmonary rehabilitation, is unsuitable for NSCLC patients with adjuvant chemotherapy after surgery. For these patients, low-volume respiratory rehabilitation (e.g., respiratory function exercise) is more suitable. LZJ exercises refer to a traditional health and fitness practice focused on controlling of the breath. LZJ is a type of respiratory function exercise widely used as a traditional rehabilitation exercise in China. LZJ exercises can regulate and control the rise and fall of Qi (vital energy) inside the body, and relate inhalation and exhalation through different mouth forms to breathe and pronounce the following sounds: “XU”, “HE”, “HU”, “SI”, “CHUI”, and “XI”. LZJ exercises contribute to balancing the energy and the functions of the inner organs.

Therefore, the objective of this study is to evaluate the efficacy and feasibility of CHM plus LZJ exercises on QoL in postoperative NSCLC patients receiving adjuvant chemotherapy. The study could also benefit from adding the additional treatment and control groups to determine whether the combination of CHM and LZJ could optimize the therapeutic effect.

## Methods/design

### Study design

**Fig. 1:**
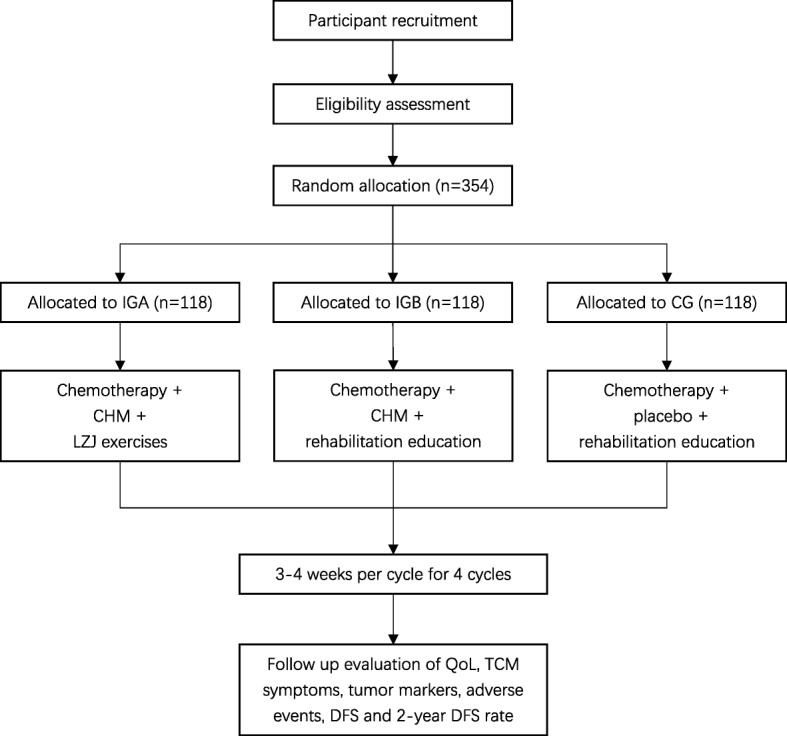
Flow diagram of the study. CG control group, CHM Chinese herbal medicine, DFS disease-free survival, IGA intervention group A, IGB intervention group B, LZJ Liu Zi Jue, QoL quality of life, TCM traditional Chinese medicine

**Fig. 2:**
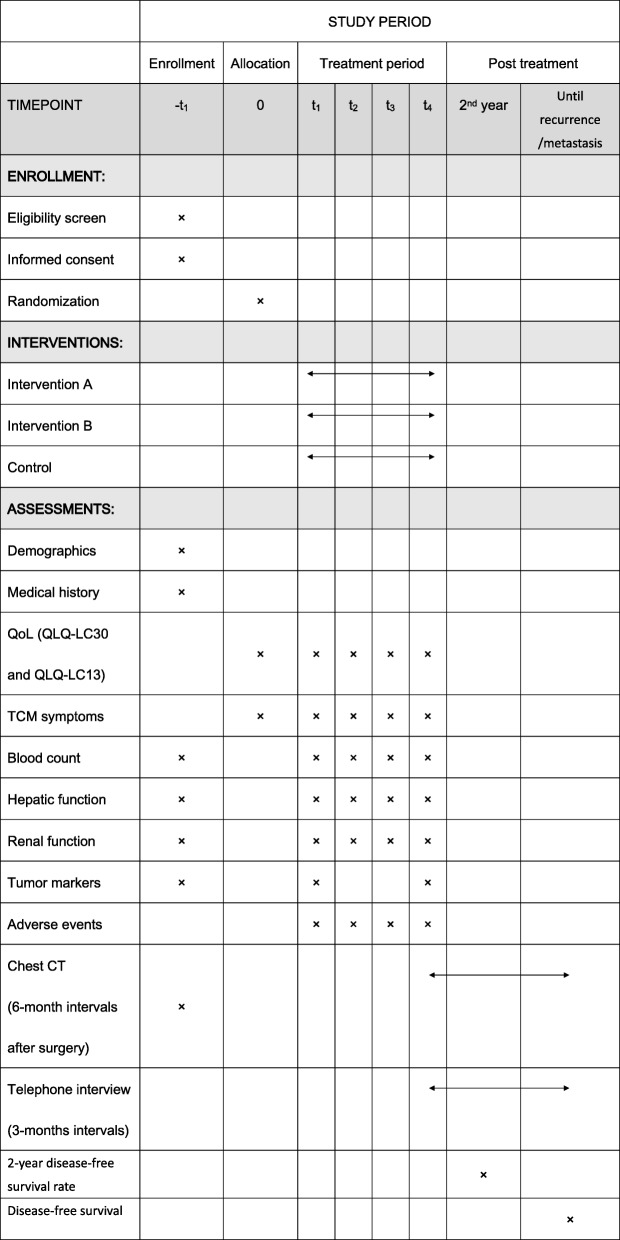
Schedule of treatment and assessment. CT computed tomography, QoL quality of life, TCM traditional Chinese medicine

This is a multicenter, randomized, controlled trial. Subjects from five clinical research centers in China will be recruited for the trial: Yueyang Hospital affiliated with Shanghai University of TCM, Shanghai Chest Hospital affiliated with Shanghai Jiao Tong University, Shanghai Pulmonary Hospital affiliated with Shanghai Tong Ji University, Shanghai Cancer Hospital affiliated with Shanghai FuDan University, and Huadong Hospital affiliated with Shanghai Fudan University. This study protocol has been approved by the Regional Ethics Review Committee of Yueyang Hospital of Integrated Traditional Chinese and Western Medicine affiliated with Shanghai University of TCM (No. 2016-059) and follows the Declaration of Helsinki. The study design is based on the SPIRIT 2013 statement [29]. Eligible participants will be randomized into three groups at a ratio of 1:1:1: intervention group A (IGA), intervention group B (IGB), and control group (CG). A flow diagram of the study is shown in Fig. 1, and the schedule of enrolment, intervention, and assessments is presented in Fig. 2.

### Study participants and recruitment

Participants in this study are NSCLC postoperative patients who will complete adjuvant chemotherapy after complete resection with a confirmed pathological diagnosis of stage Ib–IIIa. TCM syndromes include Yin deficiency, Qi deficiency, and Qi and Yin deficiency. Patients will obtain information about the study from their surgeons or recruitment brochures. The investigator will make an appointment for the clinic to assess whether the subject meets the inclusion and exclusion criteria. Eligible patients will be required to sign a written informed consent form before starting the intervention.

### Diagnostic criteria

Histologically or cytologically confirmed NSCLC, including squamous carcinoma, adenocarcinoma, adenosquamous carcinoma, and large-cell carcinoma, will be according to the Standards for the Diagnosis and Treatment of Primary Lung Cancer (2015 version) in China (National Health and Family Planning Commission of the People’s Republic of China, 2015). TNM staging of primary bronchogenic carcinoma will follow the 2009 International Anti-Cancer Alliance (UICC) staging system, version 7.

### Syndrome differentiation criteria

The syndrome differentiation criteria will follow the Clinical Practice Guidelines of Chinese Medicine in Oncology (published by People’s Medical Publishing House, National Health and Family Planning Commission of the People’s Republic of China, 2014). Symptoms will be stratified into three basic TCM syndromes and judged by two senior deputy chief physicians before treatment.

#### Qi deficiency syndrome

- Major symptoms: cough, a large amount of sputum, loss of appetite, fatigue and weakness, and a pale and bulgy tongue
- Secondary symptoms: spontaneous perspiration, loose stool, and a thin superficial and smooth pulse

#### Yin deficiency syndrome

- Major symptoms: cough, a small amount of sputum, dry mouth, and red tongue
- Secondary symptoms: night sweats, insomnia, low heat, and a thready and rapid pulse

#### Qi and Yin deficiency syndrome

- Major symptoms: cough, a small amount of sputum, fatigue and weakness, and dry mouth without polydipsia
- Secondary symptoms: spontaneous perspiration, night sweats, a reddish tongue or tongue with teeth imprints, and a thready and weak pulse

Patients with at least two main symptoms and one secondary symptom can be diagnosed.

### Inclusion criteria

The inclusion criteria are as follows: patients with completely resected stage Ib–IIIa NSCLC who will receive adjuvant chemotherapy for the first time within 6 weeks after surgery; patients aged between 18 and 74 years; patients with an Eastern Cooperative Oncology Group performance status (ECOG PS) scale of 0–2; patients without major organ dysfunction (hemoglobin ≥ 10 g/dl, absolute neutrophil count (ANC) ≥ 1.5 × 10^9^/L, and platelets ≥ 100 × 10^9^/L); patients with normal hepatic and renal functions; voluntary participation in the clinical study; and signed informed consent.

### Exclusion criteria

The exclusion criteria are as follows: indefinite pathological diagnosis; expected survival time < 6 months; heart, liver, kidney, hematopoietic system, and other serious diseases; treated with antibiotics or infected 1 week before the test; pregnant or breastfeeding women; and mental or cognitive disorders.

### Sample size calculation

The primary outcome of the study will be the change in the QoL score assessed by the QLQ-C30 scale. According to the results of the JRB.10 study, 27% of lung cancer patients at stage Ib–IIIa after 3 months of adjuvant chemotherapy had lower QoL scores than at baseline [9]. Based on the validity assumptions and clinical experience of the past, it is estimated that 15% of patients had no deterioration in the QoL combined with a comprehensive rehabilitation program compared with treatment without a comprehensive rehabilitation program for patients receiving 3-month postoperative adjuvant chemotherapy. At an inspection level of α = 0.05 and 1 – β = 0.90, the QoL score after 3 months decreased by 10% in the intervention group and by 27% in the control group compared with baseline. A sample size of 98 patients was obtained for each group, and a 20% cutoff rate was considered; therefore, 354 patients (*n* = 118) will be observed in 3 years. It is suggested that comprehensive rehabilitation combined with chemotherapy can improve the QoL of NSCLC postoperative patients compared with chemotherapy alone.

### Randomization

Stratified blocked randomization will be designed and performed by the Subject Randomization and Drug Blindness/Distribution Management System (RBS) V1.0 of Shanghai Clinical Research Center (SCRC). There will be a block size of 6, and patients will be stratified in line with the clinical stages and the center. The researcher will be able to obtain random numbers and group allocation immediately in the form of a short message service. Patients will be randomly divided into three groups at a ratio of 1:1:1.

### Blinding

This is a partially patient-blind trial. It is unavoidable that patients will know whether they are undergoing LZJ exercises or rehabilitation education. The placebo granules are indistinguishable from the CHM granules in terms of smell, color, taste, and packaging. Thus, all participants will be blinded for the medication section. A third-party independent evaluation method will be used to evaluate the outcomes. All of the outcome analyzers will be blinded to the allocation.

### Intervention

All participants will be permitted to continue their regular medications except for Chinese patent drugs. Each group will receive adjuvant platinum-based doublet chemotherapy for four cycles. IGA participants will receive chemotherapy combined with CHM and LZJ exercises, IGB participants will receive chemotherapy combined with CHM and rehabilitation education, and CG participants will receive chemotherapy combined with placebo and rehabilitation education.

Herbal treatment, CHM or placebo, will be given as granules daily for 12 weeks until the end of chemotherapy. IGA participants will perform LZJ exercises four times per week for 12 weeks. IGB and CG participants will be instructed to perform 30 min of exercises four times per week for 12 weeks. The groups are as follows:

- IGA: chemotherapy + CHM + LZJ exercises
- IGB: chemotherapy + CHM + rehabilitation education
- CG: chemotherapy + placebo + rehabilitation education

### Chemotherapy

A platinum-doublet chemotherapy regimen will be recommended for patients at stage Ib–IIIa 6 weeks after surgery. The chemotherapy will be given as four cycles in total, with every 21 days making up one cycle. One of the following five regimens will be available:

- Cisplatin 75–80 mg/m^2^ on day 1 or carboplatin AUC 5 on day 1; vinorelbine 25–30 mg/m^2^ on days 1 and 8
- Cisplatin 75–80 mg/m^2^ on day 1 or carboplatin AUC 5 on day 1; gemcitabine 1250 mg/m^2^ on days 1 and 8
- Cisplatin 75–80 mg/m^2^ on day 1 or carboplatin AUC 5 on day 1; docetaxel 75 mg/m^2^ on day 1
- Cisplatin 75–80 mg/m^2^ on day 1 or carboplatin AUC 5 on day 1; pemetrexed 500 mg/m^2^ on day 1 for nonsquamous carcinoma
- Cisplatin 75–80 mg/m^2^ on day 1 or carboplatin AUC 5 on day 1; paclitaxel 135–175 mg/m^2^ on day 1

For patients who receive fewer than the intended number of cycles of chemotherapy, the study duration will be calculated on the basis of the projected interval.

### Chinese herbal medicine and placebo

#### Chinese herbal medicine (CHM)

**Fig. 3:**
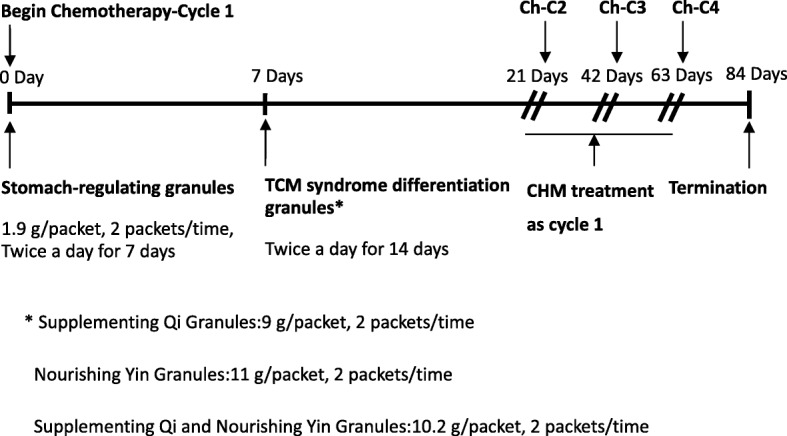
Chinese herbal medicine dosage process. *Supplementing Qi granules, 9 g/packet, 2 packets/time; nourishing Yin granules, 11 g/packet, 2 packets/time; supplementing Qi and nourishing Yin granules, 10.2 g/packet, 2 packets/time. Ch-C chemotherapy cycle, CHM Chinese herbal medicine, TCM traditional Chinese medicine

Prescriptions will be formulated into granules provided by Professor Ling Xu. Packages of granules will be made into five types of functions (e.g., stomach-regulating granules, supplementing Qi granules, nourishing Yin granules, supplementing Qi and nourishing Yin granules, and detoxifying and resolving mass granules). Each package will contain water-soluble herbal granules manufactured at a Good Manufacture Practice (GMP) standard facility (Tian Jiang Ltd, Jiangyin, China). The prescription form will comprise the stock list with both the name and serial number. Participants will take stomach-regulating granules in the first week after chemotherapy and will start to take TCM syndrome differentiation medications in the second week after chemotherapy (Fig.3).

The TCM syndrome differentiation medications will be employed based on the regimens presented in Table 1.

**Table 1:** Regimens of Chinese herbal medicine based on different syndromes

Syndrome differentiation	Chinese medicine (unit: package)			
	Supplementing Qi granules	Nourishing Yin granules	Supplementing Qi and nourishing Yin granules	Detoxifying and resolving masses granules
Qi deficiency	+	–	–	+
Yin deficiency	–	+	–	+
Qi and Yin deficiency	–	–	+	+

#### Placebo

The placebo granules will be composed of edible pigment and artificial flavors without CHM, to look and taste similar to the therapeutic granules. Placebo and the therapeutic packages will be stored in different cabinets, and only the dispensing technician will know the contents of the packages.

### Liu Zi Jue exercises and rehabilitation education

#### Liu Zi Jue (LZJ)exercises

IGA participants will learn LZJ exercises within 1 month after surgery under the leadership of a specialist at the Yue-Yang Integrative Medicine Hospital of the Shanghai University of Traditional Chinese Medicine and will perform LZJ exercises four times per week at home during adjuvant chemotherapy.

The program of LZJ exercises includes the following: warm-up, patients will perform 5 min of joint activities; LZJ exercises, patients will exhale through six different mouth forms to breathe and pronounce the sounds “XU”, “HE”, “HU”, “SI”, “CHUI”, and “XI” in turn with corresponding actions for approximately 30 min in total; and relaxation, patients will adjust their breathing and relax their muscles for 5 min.

#### Rehabilitation education

IGB and CG participants will be instructed to perform 30 min of exercises four times per week that could maintain their physical strength (e.g., deep breathing training, slow walking, and jogging) during chemotherapy.

### Outcome measurement

#### Primary outcome

- Quality of life: assessed with the EORTC QOL-LC43. The scoring method will be used to determine the results based on the score changes. Before and after the intervention, the international quality scoring system will be used to calculate the scores of general QoL and various fields.

#### Secondary outcomes

- Two-year disease-free survival rate: this rate refers to the percentage of patients without recurrence and metastasis within 2 years after surgery.
- Disease-free survival (DFS): DFS refers to the interval time from the date of randomization to either the date of the first documented progression or the date of death from any cause, whichever comes first.
- TCM symptom changes: TCM symptom scores will be recorded and calculated based on the grading scale of lung cancer symptoms required in The Guiding Principles of Clinical Research of New Chinese Medicine treating Primary Bronchial Lung Cancer (2002) issued by National Medical Products Administration. The changes before and after each treatment will be applied for the assessment of efficacy.
- Physical condition: the physical condition of the patient will be assessed following the ECOG PS standard before and after treatment.
- Tumor markers: tumor markers, which include CEA, CA-125, and CYFRA21-1, will be measured before and after treatment.
- Safety and adverse events: according to the Common Terminology Criteria for Adverse Events (CTCAE) V4.0 issued by the National Cancer Institute (NCI) (https://ctep.cancer.gov), all of the patients will be assessed before and after treatment. The evaluation includes hematological and nonhematological adverse events. Complete blood count, hepatic and renal functions, urine and stool routine tests, and electrocardiograms will be measured to assess hematological toxicity. Other adverse events, including toxicity and side effects, of each group will also be recorded and reported during treatment. If there is a serious adverse event (SAE), the treatment will be stopped immediately, and appropriate treatment will be provided. The types and frequencies of adverse events in each group will be reported.

### Data collection and monitoring

The researchers from each center will collect their data with the use of a Case Report Form (CRF). SCRC will be responsible for establishing a special database for data entry and management. Two data managers will independently input and proofread the data. Specialists will check the data for each center every month. Relevant researchers will need to verify and correct any problems found within 1 week.

### Statistical analysis

The data will be accessed and saved into the project database of SCRC. SAS software will be applied for statistical analysis after access. The methods of statistical analysis are as follows.

A paired *t* test will be performed to compare the average score before and after treatment in each group, and an independent-sample *t* test will be performed to compare the scores between the two groups. According to the homogeneity of variance test, a rank-sum test between two groups will be performed for those whose *P* value is not consistent with a normal distribution. A chi-squared test will be used for variable data (baseline, medicine for adjuvant chemotherapy). A rank-sum test will be performed to analyze ordered hierarchical data (NCI CTC-graded adverse events). The Kaplan–Meier method will be employed to analyze the median survival time of the two groups. Log-rank will be used to test the median survival time. Statistical significance will be defined as *P* < 0.05 with a two-sided test.

### Quality control

Researchers must be trained in Good Clinical Practice for those who have the expertise, qualifications, and competence to participate in clinical trials. All medical staff will be uniformly trained before the start of the project so that they have a full understanding of the clinical trial. Each researcher will be required to have an Investigator’s Brochure for easy access. All data quality control will be carried out every 3 months, and the problems of supervision and inspection will be corrected within 48 h after the end of the quality control.

### Confidentiality

Only researchers involved in clinical trials may be exposed to and confidential with the subject’s personal medical records. The personal information of the identifiable subject will be omitted anonymously during data processing.

## Discussion

Postoperative adjuvant therapy has been a useful attempt in recent years, and several studies have revealed that EGFR-TKI targeted therapy can effectively extend the recurrence and metastasis time of patients with sensitive mutations after surgery [30]. However, adjuvant chemotherapy remains the preferred treatment for postoperative NSCLC patients as the follow-up strategy for drug resistance to targeted therapy has not been clarified. The toxicity and side effects of chemotherapy not only affect the QoL of patients but also decrease the completion rate of treatment. As a part of integrated medicine, TCM has been vital for improving the efficacy and reducing the toxicity of chemotherapy. In previous studies, it has been preliminarily verified that adjuvant chemotherapy (NP/NC) combined with CHM in NSCLC patients after resection improves the QoL compared with chemotherapy alone. TCM also enables patients to complete adjuvant chemotherapy more safely and effectively. In this study, the selection of chemotherapy regimens is enriched, all of the adjuvant chemotherapy regimens commonly used in postoperative patients with NSCLC are included, and the treatment of CHM interventions is adjusted. To improve the patients’ digestive tract reaction within 1 week after chemotherapy and avoid the nausea and vomiting caused by taking CHM, TCM granules are to be suspended during chemotherapy, and participants in the intervention group will take stomach-regulating granules in the first week after chemotherapy. Patients will begin to take TCM syndrome differentiation medications from the recovery period of chemotherapy to recover their physical strength and prepare for the next cycle of chemotherapy. The inclusion and exclusion criteria will improve the homogeneity of baseline values, which are more conducive to quality control criteria.

The aim of this study is to further prove whether chemotherapy combined with a comprehensive rehabilitation program based on CHM and LZJ exercises can improve the QoL and prolong patient survival. A placebo-controlled double-blind RCT design and protocol are proposed, which provides evidence for the QoL in patients with NSCLC after chemotherapy combined with or without a comprehensive rehabilitation program. The results will support comprehensive treatment of patients with NSCLC.

### Trial status

Protocol version 2.0 was finished on September 13, 2016. Participant recruitment started in October 2016 and is expected to end in February 2020.

## Data Availability

As the research has not yet been completed, the datasets generated and analyzed during the current study are not publicly available but are available from the corresponding author upon reasonable request.

## Abbreviations

ANC: Absolute neutrophil count
CG: Control group
CHM: Chinese herbal medicine
CRF: Case Report form
CTCAE: Common Terminology Criteria for Adverse Events
DFS: Disease-free survival
ECOG PS: Eastern Cooperative Oncology Group performance status
EORTC: European Organization for Research and Treatment of Cancer
GMP: Good Manufacture Practice
IGA: Intervention group A
IGB: Intervention group B
LZJ: Liu Zi Jue
NCI: National Cancer Institute
NSCLC: Nonsmall-cell lung cancer
QoL: Quality of life
SAE: Serious adverse event
SCRC: Shanghai Clinical Research Center
TCM: Traditional Chinese medicine
